# The renin-angiotensin system modulates endotoxic postconditioning of exacerbated renal vasoconstriction in preeclamptic offspring

**DOI:** 10.1038/s41598-023-27923-7

**Published:** 2023-01-17

**Authors:** Hagar A. Morgaan, Marwa Y. Sallam, Hanan M. El-Gowelli, Sahar M. El-Gowilly, Mahmoud M. El-Mas

**Affiliations:** 1 Department of Pharmacology and Toxicology, Faculty of Pharmacy, Alexandria University, Alazarita, Alexandria, 21521 Egypt; 2 Department of Pharmacology and Toxicology, Faculty of Medicine, Kuwait University, Kuwait City, Kuwait

**Keywords:** Developmental biology, Diseases, Nephrology, Pathogenesis

## Abstract

We recently reported exacerbated endotoxic signs of neuroinflammation and autonomic defects in offspring of preeclamptic (PE) dams. Here, we investigated whether PE programming similarly modifies hemodynamic and renal vasoconstrictor responsiveness to endotoxemia in PE offspring and whether this interaction is modulated by gestational angiotensin 1–7 (Ang1-7). Preeclampsia was induced by gestational treatment with L-NAME. Adult offspring was challenged with lipopolysaccharides (LPS, 5 mg/kg) and systolic blood pressure (SBP) and renal vasoconstrictions were assessed 4 h later. Male, but not female, offspring of PE rats exhibited SBP elevations that were blunted by LPS. Renal vasoconstrictions induced by angiotensin II (Ang II), but not phenylephrine, were intensified in perfused kidneys of either sex. LPS blunted the heightened Ang II responses in male, but not female, kidneys. While renal expressions of AT1-receptors and angiotensin converting enzyme (ACE) were increased in PE offspring of both sexes, ACE2 was upregulated in female offspring only. These molecular effects were diminished by LPS in male offspring. Gestational Ang1-7 caused sex-unrelated attenuation of phenylephrine vasoconstrictions and preferentially downregulated Ang II responses and AT1-receptor and nuclear factor-kB (NFkB) expressions in females. Together, endotoxemia and Ang1-7 offset in sexually-related manners imbalances in renal vasoconstriction and AT1/ACE/ACE2 signaling in PE offspring.

## Introduction

Preeclampsia (PE) is an alarming life-threatening obstetric disorder manifested as high blood pressure > 140/90 after 20 weeks of gestation together with proteinuria and other systemic manifestations^1^. The prevalence of PE is estimated to be 2–8% of all pregnancies^2^. Clinical and experimental data indicate that PE does not only jeopardize maternal and perinatal health, but also predispose offspring to serious complications later in life, a phenomenon usually referred to as preeclamptic fetal programming^3–5^. Offspring born to preeclamptic dams are at higher risk of developing renal^6,7^ and cardiovascular complications^8,9^ during adulthood.

Alternatively, endotoxemia is a life-threatening clinical syndrome in which exaggerated immune response to infection results in multiple organ dysfunction^10,11^. Lipopolysaccrides (LPS), a major component of gram negative bacterial cell wall, is commonly used to induce experimental endotoxemia and model the hyperinflammatory state of early sepsis^12,13^. LPS induces endotoxic shock via upregulation of toll-like receptor 4 and subsequent activation of NFkB and other inflammatory mediators^14–16^. Acute kidney injury and vascular hyporeactivity are common complications of endotoxic shock^17,18^. We and others have reported sex-related discrepancies in inflammatory response to endotoxemia, with females being less susceptible than male subjects^13,19,20^.

In addition to its role in regulating blood pressure and electrolyte balance, RAS is thought to contribute to inflammatory disorders^21^. RAS modulation of physiological and pathophysiological states are mediated via 2 major arms, the pressor (Ang II/ACE/AT_1_ receptors) and depressor arms (Ang1-7/ACE2/Mas receptors)^22^. Contradictory reports are available regarding the roles of RAS components in PE pathophysiology. Compared with uncomplicated pregnancies, humans and experimental studies showed that while PE is often associated with diminished blood renin and Ang II, vascular expression of AT1 receptors as well as vasoconstrictor and inflammatory actions of Ang II are exaggerated^23–26^. Moreover, published data on ACE activity and expression during PE are contradictory^26^. Alternatively, Ang1-7 signaling is depressed over the course of PE^27^ and gestational Ang1-7 supplementation ameliorates the PE-related hypertension, inflammation, and oxidative stress through the upregulation of peroxisome proliferator-activated receptors gamma^28^. Similar favorable effects for Ang1-7 against inflammatory and cardiovascular signs of endotoxemia have been reported^29,30^.

Although PE in humans and experimental animals, including L-NAME model, is often coupled with reduced levels of circulating renin and Ang II, vascular AT1 receptor expression and vasoconstrictor and inflammatory actions of the peptide are intensified compared with those with uncomplicated pregnancies^23–26^. On the other hand, reported data on ACE activity and expression during PE are contradictory^26^.

In a recent study, we provided the first experimental evidence that PE fetal programming enhances the vulnerability of adult offspring to cardiovascular and autonomic neuropathic sequels of endotoxemia^3^. Such priming potential of PE appeared in male but not female offspring and was provoked by exacerbated myocardial and brainstem inflammatory signals^3^. That said, it is not clear whether renal homeostasis in PE offspring could be reprogrammed in a similar manner. In this study, we tested the hypotheses that (i) preeclamptic fetal programming refashions renal vasoconstrictor and hemodynamic profiles of endotoxemia in adult offspring, (ii) fetal reprogramming is influenced by the offspring sex and gestational Ang1-7 supplementation, and (iii) molecular entities of the ACE/ACE2/AT1 receptor pathway mediate the PE-LPS interactions.

## Results

Table 1 shows body and kidney weights of adult offspring of various control (non-PE) and PE dams. There were no statistically significant differences in these parameters in adult rats of the same sex. On the other hand, body and kidney weights in female offspring were consistently and significantly smaller than respective values in male counterparts.

**Table 1:** Values of body and kidney weights (in grams) in adult offspring of control (non-PE) and PE rats.

Rat group	Male rats		Female rats	
	Body weight	Kidney weight	Body weight	Kidney weight
Control	226 ± 7	0.918 ± 0.028	190 ± 5*	0.758 ± 0.025*
PE	205 ± 7	0.917 ± 0.025	178 ± 5*	0.757 ± 0.029*
LPS	221 ± 6	0.923 ± 0.029	189 ± 7*	0.755 ± 0.034*
PE/LPS	203 ± 5	0.922 ± 0.029	180 ± 4*	0.762 ± 0.022*
PE/Ang1-7/LPS	207 ± 10	0.915 ± 0.014	183 ± 6*	0.752 ± 0.019*

Values are presented as means ± SEM of 6–8 observations.

*P < 0.05 vs. respective values in male rats.

### Effect of PE and/or LPS on SBP in adult offspring

**Figure 1:**
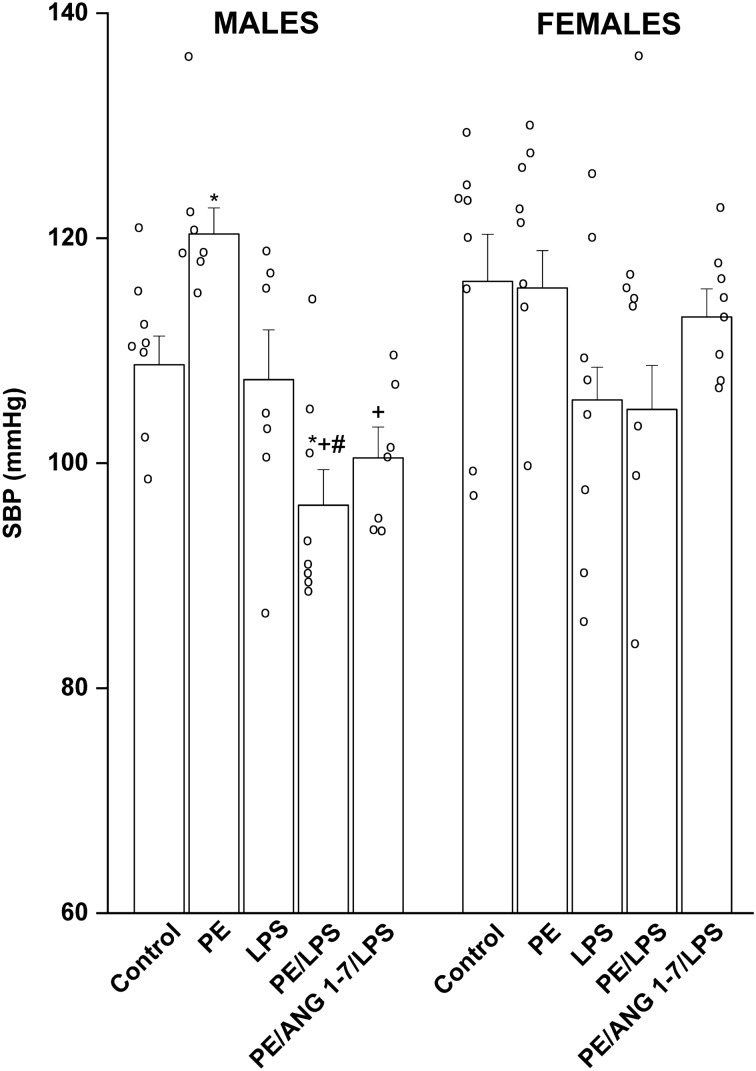
Tail cuff systolic blood pressure (SBP) and heart rate (HR) measurements 4 h post intraperitoneal injection of LPS (5 mg/kg) in adult male and female offspring of preeclamptic (PE) or non-PE dams. The influences of prenatal administration of Ang1-7 on SBP an HR are also shown. Values are expressed as means ± S.E.M of 6–8 measurements. The One-way ANOVA followed by the Tukey's post hoc was utilized to measure statistical significance. *P < 0.05 vs. “Control”, ^+^P < 0.05 vs. “PE”, ^#^P < 0.05 vs. “LPS” in the same sex.

Figure 1 illustrates the PE programming effect on SBP in adult offspring in the absence and presence of the endotoxic challenge. Tail-cuff measurements demonstrated that male offspring of PE rats exhibited significant rises in SBP compared with respective males of non-PE dams. While the 4-h exposure of male offspring of non-PE rats to LPS (5 mg/kg) caused no changes in SBP, significant falls in SBP were noted when the same LPS dose was administered to preeclamptic male offspring, highlighting the ability of LPS to abolish the preeclamptic rise in SBP (Fig. 1). Such dramatic decrements in SBP remained unaltered in offspring of PE/LPS rats treated gestationally with Ang1-7 (576 µg/kg/day for 7 days). On the other hand, no significant alterations in SBP were observed in female offspring in response to separate or combined PE and LPS interventions. The treatment with LPS or Ang1-7 caused slight reductions in SBP that were not statistically different from corresponding values in female offspring of non-PE rats (Fig. 1).

### Effect of PE and/or LPS on renal vasoconstriction in adult offspring

**Figure 2:**
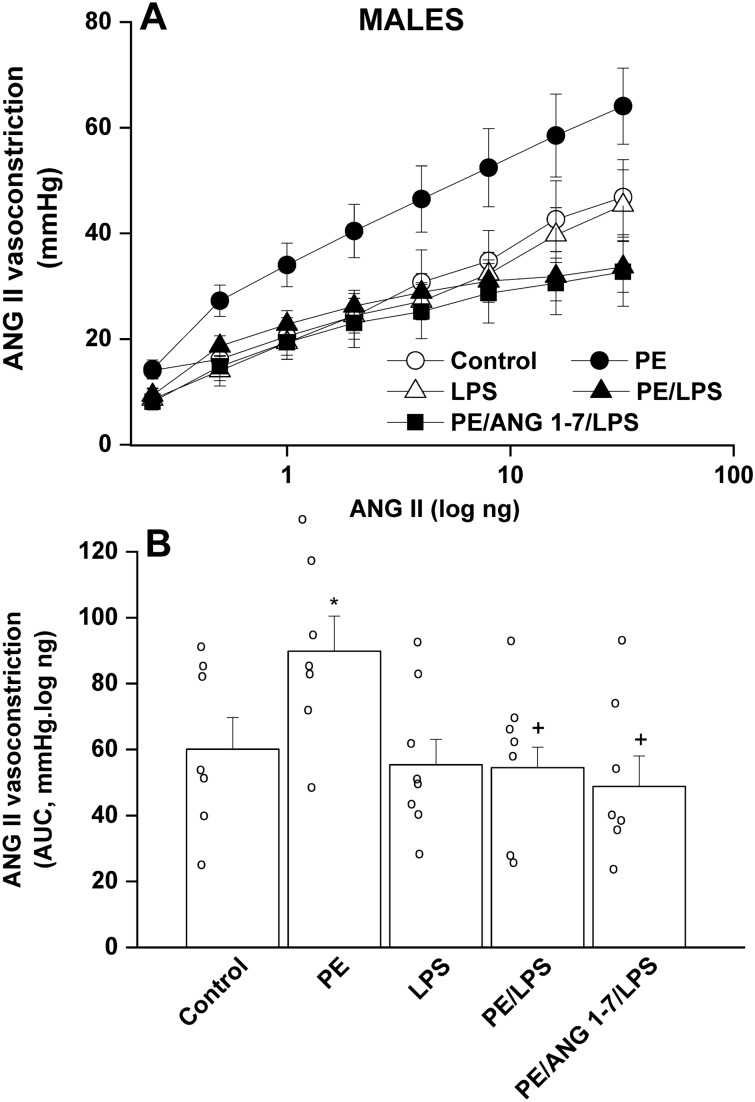
Effect of PE programming on cumulative vasoconstrictor response curves of Ang II (**A**) and AUCs of the Ang II cumulative vasoconstrictor response curves (**B**) measured 4 h post LPS challenge (5 mg/kg) in isolated perfused kidneys of adult male offspring. The influence of prenatal administration of Ang1-7 is also shown. Contractile responses were estimated as changes from basal renal perfusion pressure. Values are expressed as means ± S.E.M of 7–8 measurements. ANOVA followed by the Tukey's post hoc was utilized to measure statistical significance. *P < 0.05 vs. “Control”, ^+^P < 0.05 vs. “PE” in the same sex.

**Figure 3:**
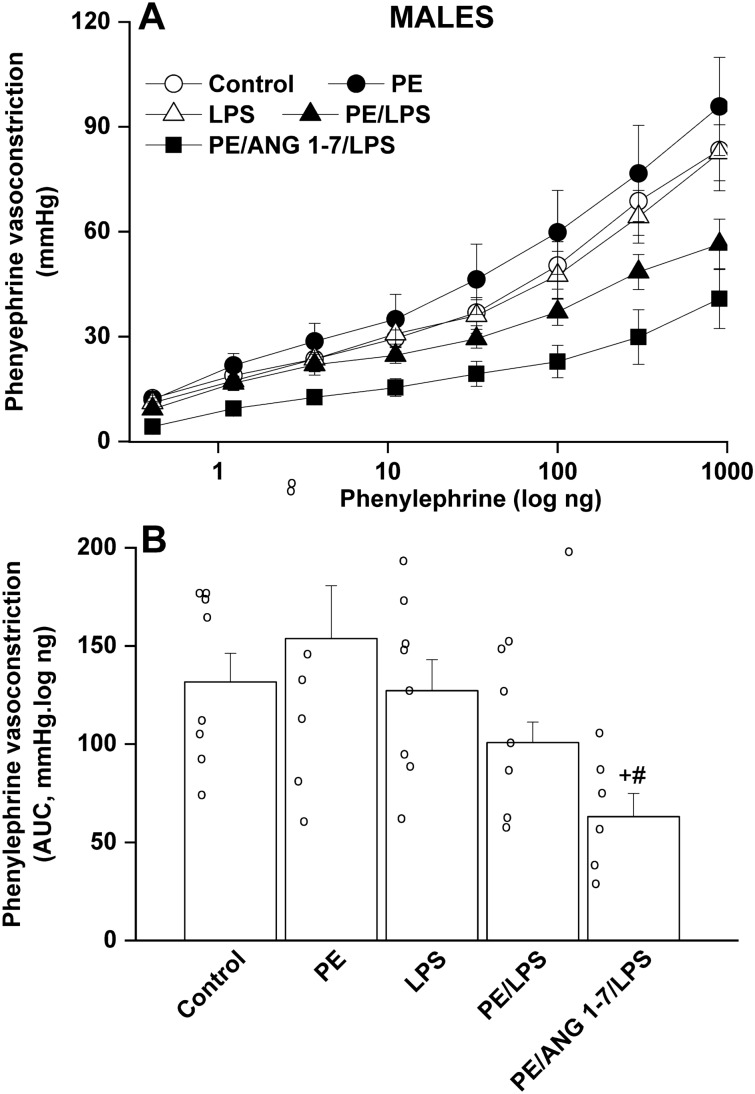
Effect of PE programming on the cumulative vasoconstrictor response curves of phenylephrine (**A**) and AUCs of the phenylephrine cumulative vasoconstrictor response curves (**B**) measured 4 h post LPS challenge (5 mg/kg) in isolated perfused kidneys of adult male offspring. The influence of prenatal administration of Ang1-7 is also shown. Contractile responses were estimated as changes from basal renal perfusion pressure. Values are expressed as means ± S.E.M of 7–8 measurements. ANOVA followed by the Tukey's post hoc was utilized to measure statistical significance. ^+^P < 0.05 vs. “PE”, ^#^P < 0.05 vs. “PE/LPS” in the same sex.

**Figure 4:**
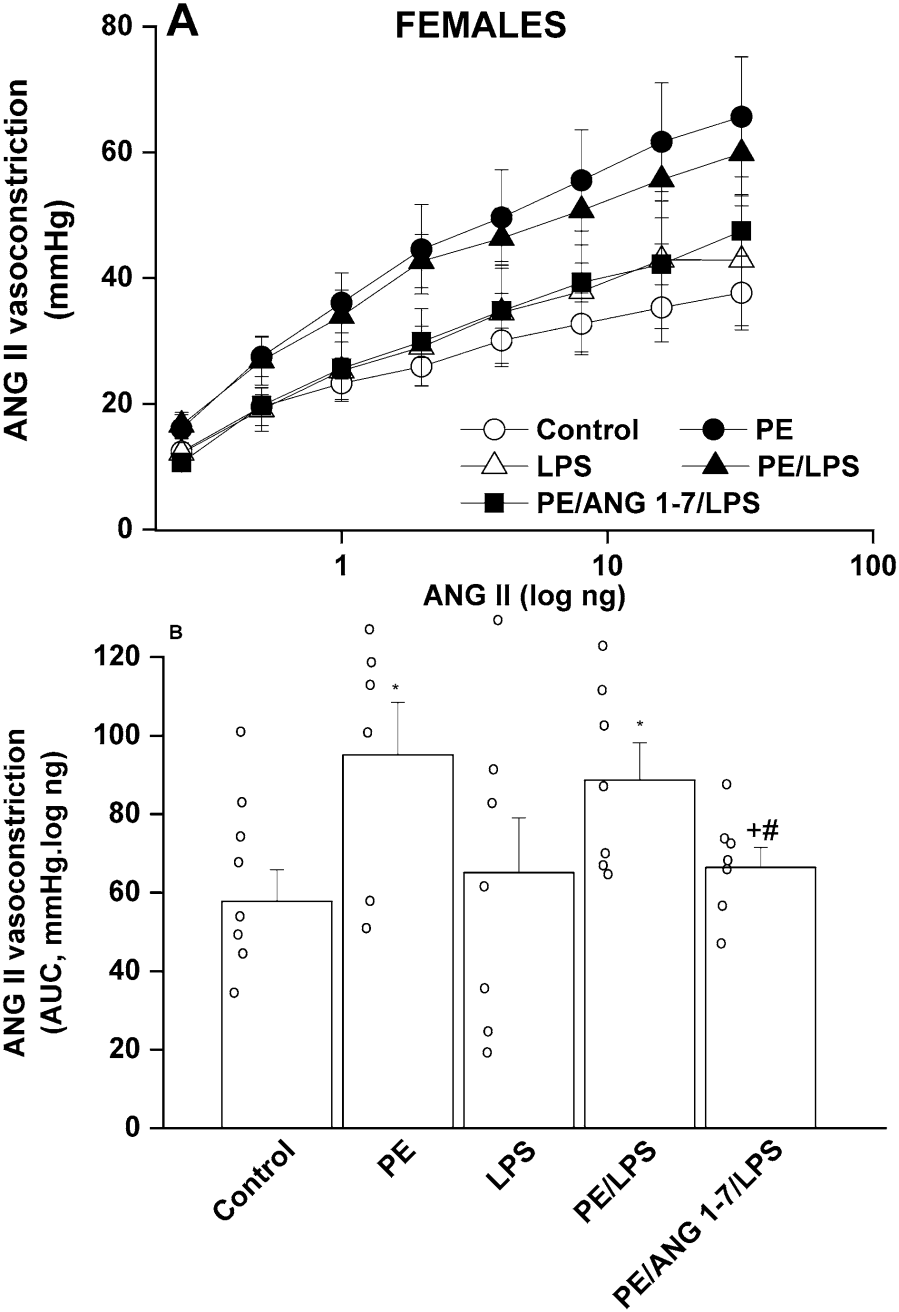
Effect of PE programming on cumulative vasoconstrictor response curves of Ang II (**A**) and AUCs of the Ang II cumulative vasoconstrictor response curves (**B**) measured 4 h post LPS challenge (5 mg/kg) in isolated perfused kidneys of adult female offspring. The influence of prenatal administration of Ang1-7 is also shown. Contractile responses were estimated as changes from basal renal perfusion pressure. Values are expressed as means ± S.E.M of 7–8 measurements. ANOVA followed by the Tukey's post hoc was utilized to measure statistical significance. *P < 0.05 vs. “Control”, ^+^P < 0.05 vs. “PE”, ^#^P < 0.05 vs. “PE/ LPS” in the same sex.

**Figure 5:**
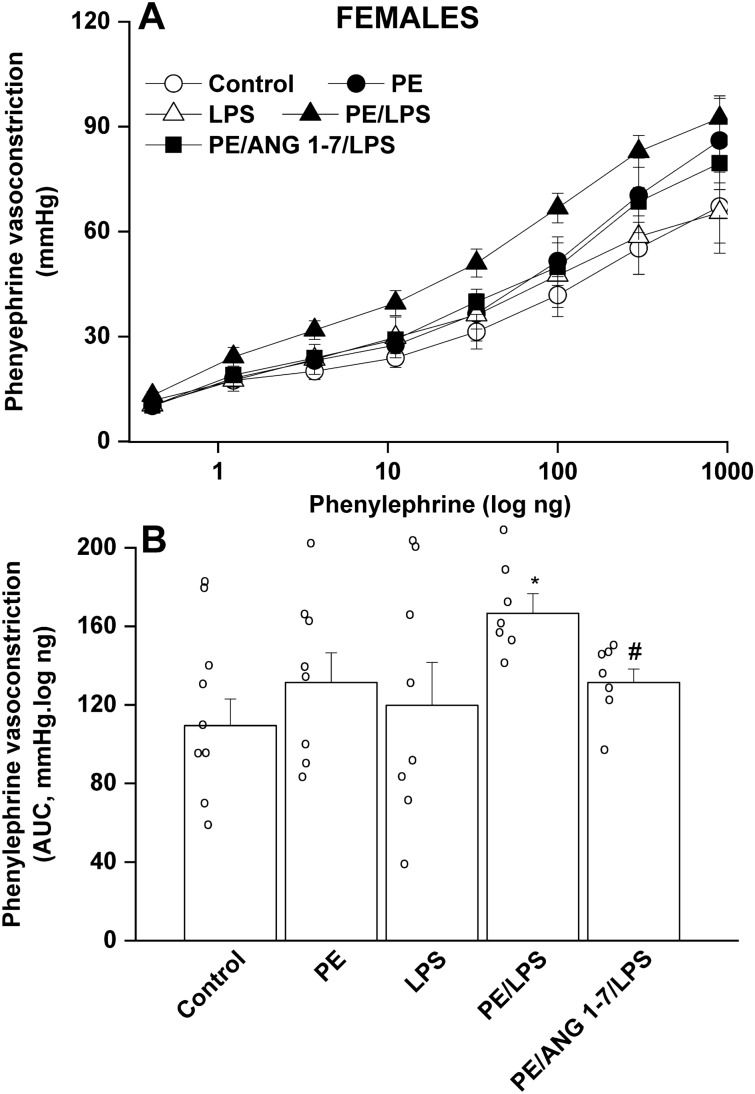
Effect of PE programming on the cumulative vasoconstrictor response curves of phenylephrine (**A**) and AUCs of the phenylephrine cumulative vasoconstrictor response curves (**B**) measured 4-h post LPS challenge (5 mg/kg) in isolated perfused kidneys of adult female offspring. The influence of prenatal administration of Ang1-7 is also shown. Contractile responses were estimated as changes from basal renal perfusion pressure. Values are expressed as means ± S.E.M of 7–8 measurements. ANOVA followed by the Tukey's post hoc was utilized to measure statistical significance. *P < 0.05 vs. “Control”, ^#^P < 0.05 vs. “PE/ LPS” in the same sex.

Isolated perfused kidneys were used to evaluate renal vasoconstrictor responsiveness to cumulative doses of Ang II (0.25–32 ng) and phenylephrine (0.41–900 ng). The cumulative vasoconstrictor effects of Ang II and phenylephrine were assessed by calculating the area under the curve (AUC) for individual experiments. PE male offspring kidneys exhibited heightened vasoconstrictions to Ang II (Fig. 2), but not phenylephrine (Fig. 3), compared with their respective non-PE groups. LPS had no effect on Ang II responses, but virtually abolished the potentiated Ang II vasoconstrictions in male rats (Fig. 2). In female offspring, intensified Ang II responses were noted in the PE female group but contrary to males, these elevated Ang II responses remained unaltered following endotoxic insult in PE/LPS group (Fig. 4). Moreover, the vasopressor effects of phenylephrine were not affected by PE or LPS, but showed significant increases in PE/LPS female kidneys (Fig. 5).

In PE/LPS rats treated prenatally with Ang1-7, significant reductions in phenylephrine (Fig. 3), but not Ang II (Fig. 2), responses were demonstrated in male offspring compared with the PE/LPS insult. On the other hand, the PE/LPS-evoked potentiation in Ang II (Fig. 4) and phenylephrine vasoconstrictions (Fig. 5) in female offspring were eliminated by gestational Ang1-7.

### Roles of RAS and NFkB signaling in PE/LPS interaction

**Figure 6:**
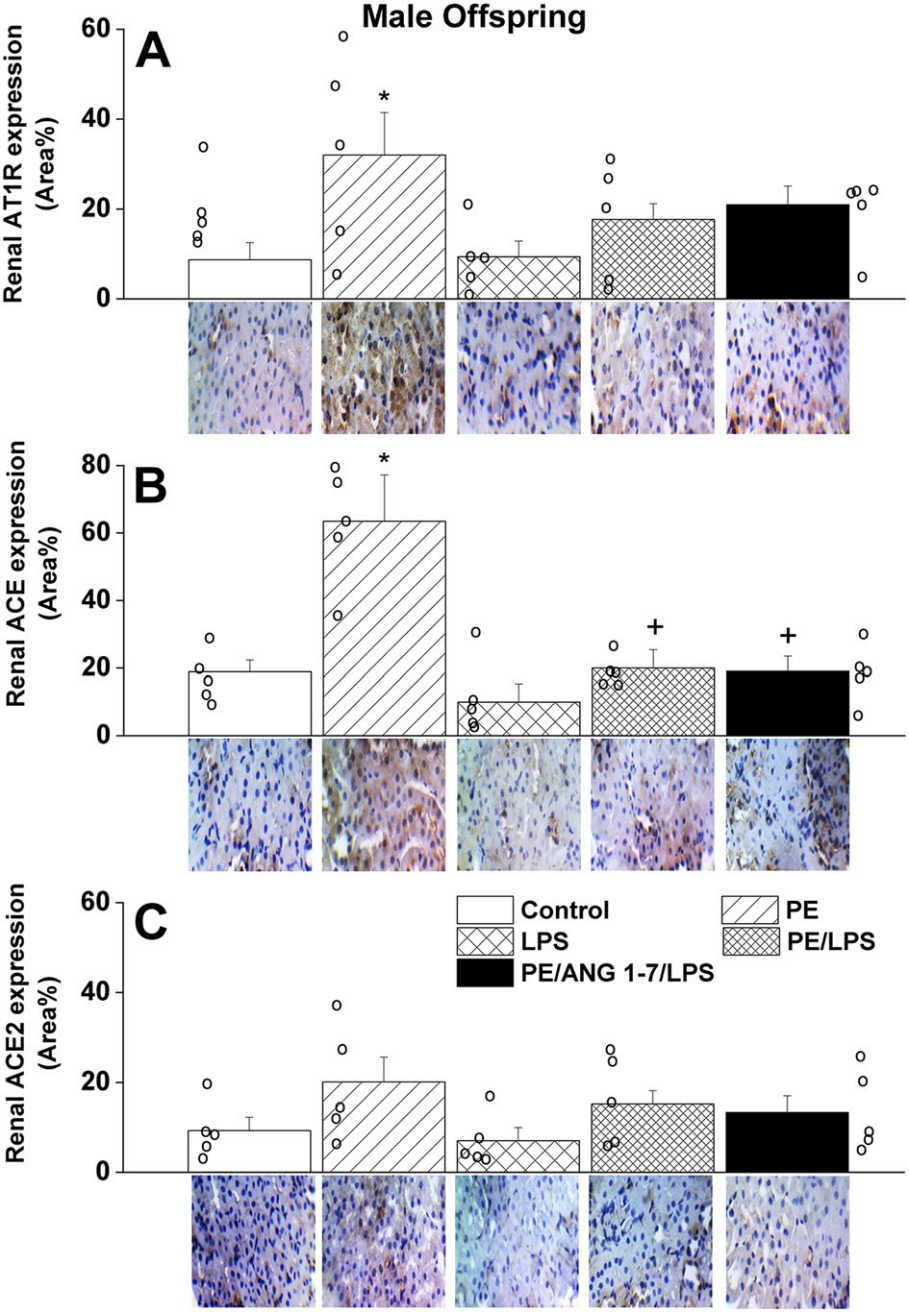
Effect of PE and/or LPS on immunohistochemical protein expression of AT1 receptors (**A**), ACE (**B**) and ACE2 (**C**) in renal tubular tissues of male offspring. The influence of prenatal administration of Ang1-7 is also shown. The One-way ANOVA followed by the Tukey's post hoc was utilized to measure statistical significance. Values are expressed as means ± S.E.M of 4–5 observations. *P < 0.05 vs. “Control”, ^+^P < 0.05 vs. “PE” in the same sex. Representative images for immunostained sections from the renal tubular tissues are also shown.

**Figure 7:**
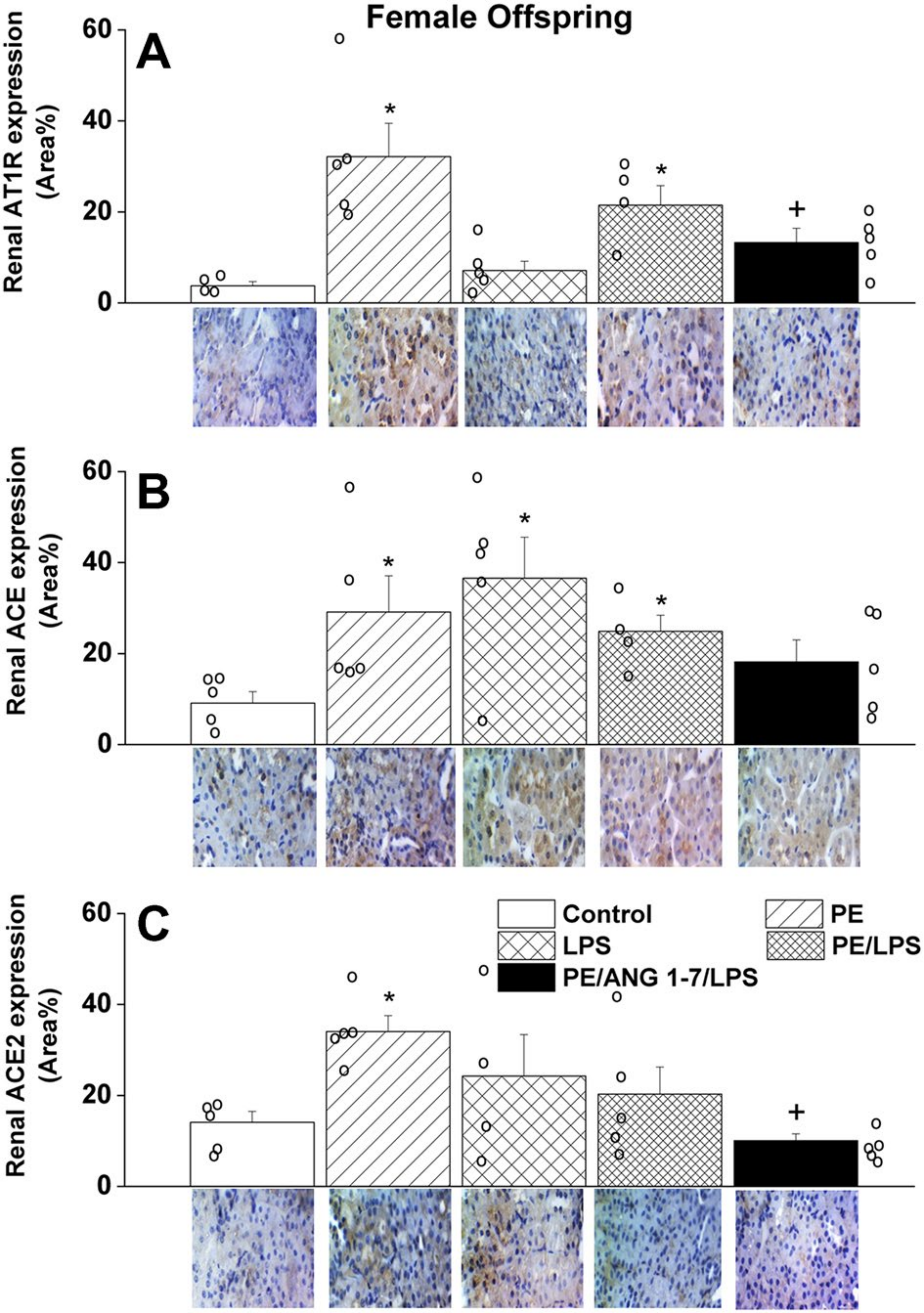
Effect of PE and /or LPS on immunohistochemical protein expression of AT1 receptors (**A**), ACE (**B**) and ACE2 (**C**) in renal tubular tissues of female offspring. The influence of prenatal administration of Ang1-7 is also shown. The One-way ANOVA followed by the Tukey's post hoc was utilized to measure statistical significance. Values are expressed as means ± S.E.M of 4–5 observations. *P < 0.05 vs. “Control”, ^+^P < 0.05 vs. “PE” in the same sex. Representative images for immunostained sections from the renal tubular tissues are also shown.

Compared with control (non-PE) offspring, immunohistochemical studies revealed significant increases in renal tubular expressions of molecular entities of the offensive RAS arms, AT1 receptors (Figs. 6A and 7A) and ACE (Figs. 6B and 7B), in both PE male and female offspring. However, significantly higher expression levels of ACE expression were noted in kidneys of male compared with female offspring (63 ± 13 vs. 29 ± 7 area%, p < 0.05). These PE effects were eliminated in LPS-challenged male rats but remained manifest in female rats (Figs. 6 and 7). The treatment of control non-PE rats with LPS had no effect on AT1 receptor or ACE expressions, except probably for a significant rise in ACE expression in female rats only. Alternatively, the tubular expression of ACE2, the defensive arm of RAS, was increased in female (Fig. 7C), but not male (Fig. 6C), offspring of PE dams and this effect was depressed after LPS treatment and more so following gestational Ang1-7 administration. Moreover, the significant rises in tubular AT1 receptor expression seen in female offspring of PE or PE/LPS dams disappeared after Ang1-7 supplementation (Fig. 7A).

**Figure 8:**
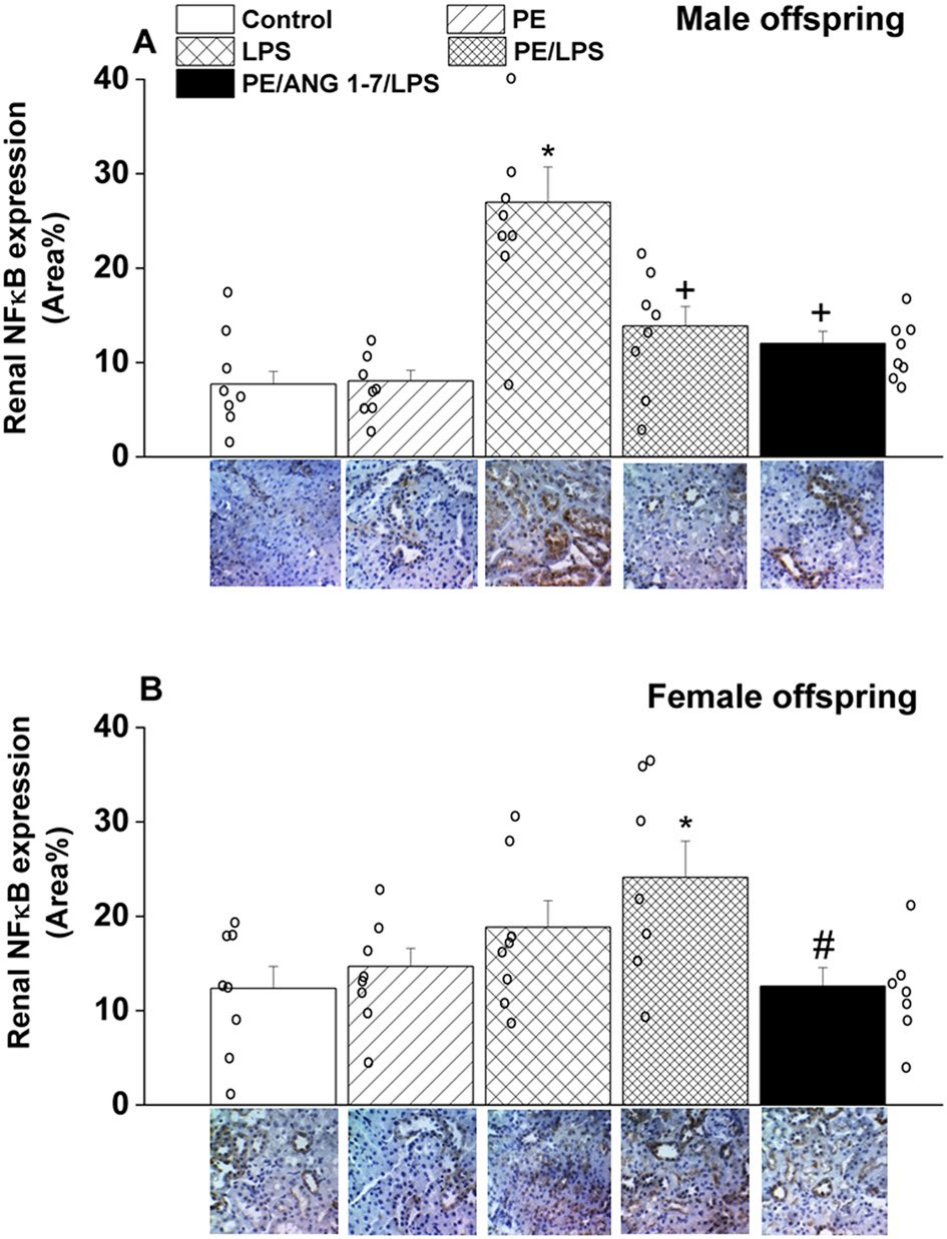
Effect of PE and /or LPS on immunohistochemical protein expression of NFκB in renal tubular tissues of male (**A**) and female offspring (**B**). The influence of prenatal administration of Ang1-7 is also shown. The One-way ANOVA followed by the Tukey's post hoc was utilized to measure statistical significance. Values are expressed as means ± S.E.M of 6–7 observations. *P < 0.05 vs. “Control”, ^+^P < 0.05 vs. “LPS”, #P < 0.05 vs. “PE/LPS” values. Representative images for immunostained sections from the renal tubular tissues are also shown.

Figure 8 shows that tubular NFkB expression was also altered in a sexually differentiated manner. LPS significantly increased tubular NFkB expression in male offspring of control dams, but not in PE dams with or without gestational Ang1-7 treatment (Fig. 8A). By contrast, a significant rise in NFkB expression was noted only in offspring of PE/LPS dams and this effect disappeared upon gestational treatment with Ang1-7 (Fig. 8B).

## Discussion

This study reports on the effects of PE fetal programming and prenatal Ang1-7 supplementation on renal inflammation and vasoconstrictor anomalies induced by endotoxemia in adult rat offspring. The data showed that challenging male, but not female, offspring with LPS abolished the PE-mediated elevations in SBP and interrelated renal Ang II vasoconstriction. Whereas protein renal tubular ACE/AT1 receptor expressions were upregulated in both sexes, the defensive ACE2 expression was preferentially enhanced in females only. These molecular effects disappeared by LPS in males but not females. Antenatal Ang1-7 exposure (i) had no effect on SBP but diminished the heightened renal vasoconstrictions in both sexes, and (ii) normalized the altered AT1/ACE/ACE2 and NFκB signaling in female offspring. The data suggest sexually dimorphic conditioning effects for endotoxemia and Ang1-7 on renal vasoconstrictor/inflammatory irregularities induced by PE in adult offspring.

The consequences of PE often extend beyond conception predisposing offspring to serious complications during adulthood. Our data demonstrated an elevated SBP that was evident only in male offspring of PE mothers. Such pattern of sexual dimorphism in response to in utero fetal insult is consistent with previous studies and suggest a pivotal role for gender in determining the BP phenotype following prenatal insults^3,31–34^. Alternatively, the 4-h exposure of male and female offspring of non-PE rats to LPS caused no changes in SBP. Of note, reported hemodynamic responses following LPS administration are variable with some studies showing a decrease in BP^35–38^, while others demonstrating no changes^39,40^, or even elevations in BP^41^. Such controversy in the BP response to LPS may be related to differences in rat strain, dose of LPS, route of administration and duration of exposure. Lee et al.^42^ and Mehanna et al.^35^ demonstrated 3 distinct phases in BP during time course of endotoxemia, an initial hypotensive response phase that is followed by a rebound recovery in BP and then a long-lasting hypotensive state. Alterations in baroreflex sensitivity and peripheral resistance^41^ and imbalances in pro-inflammatory/anti-inflammatory cytokine profiles over the time course of sepsis^43^ may account for such effects.

In contrast to its effects in offspring of non-PE rats, LPS administration to male offspring of PE dams produced significant falls in BP. Such vulnerability of male offspring of PE rats to LPS hypotension supports the hypothesized role of PE fetal programming in the escalated incidence of cardiovascular complications during adulthood. The lack of such effect in the female offspring is consistent with previous clinical^44,45^ and experimental studies^3,38,46^, which suggested a protective role for female sex hormones against endotoxic cardiovascular insults. The latter view is reinforced by the the findings that bilateral ovariectomy^47^ or pharmacologic blockade of gonadal hormone receptors^12,13^ uncover clear hypotensive and inflammatory responses to endotoxemia. Notably, the tail‐cuff technique employed in the current study for SBP measurement is believed to suffers some inherent limitations such as the need for preheating and restraining of the animal, which may undermine the reliability of the results^48^. To minimize the impact of these stressors, rats were subjected to daily pre-conditioning for at least 3 consecutive days before the actual measurement of SBP. Moreover, SBP was measured in triplicates and values were averaged. Figure 1 shows that the data points of SBP are mostly tightly clustered around the mean values.

One basic objective of the current study was to investigate how the offspring renal vasoconstrictor propensity could be influenced by PE/LPS interaction. Reported data on the effect of PE or fetal hypoxic programming on renal vasoconstriction in the offspring adulthood are rare and inconsistent. Evidence suggests that preeclamptic hypoxic conditions (i) augment placental expression of hypoxia-inducible factor 1-alpha and causes imbalances in angiogenic and antiangiogenic machineries^49,50^, and (ii) exacerbate α_1_-adrenoceptor^51^ and Ang II receptor-mediated vasoconstriction^52^ in renal and cerebral arteries of male offspring, respectively. The upregulated hypoxic insult and RAS signaling during PE negatively correlate and perhaps predispose to vascular endothelial dysfunction^53^. On the other hand, Williams et al.^54^ reported opposite changes in phenylephrine vasoconstriction in carotid and femoral arteries of offspring exposed to prenatal hypoxia or nutrient restriction. The altered vasoconstrictor response might be a compensatory in-utero mechanism that is preserved beyond conception^54^. In the current study, we showed exaggerated renal vasoconstrictions to Ang II, but not phenylephrine, in both male and female PE offspring.

Intriguingly, the current study revealed a weakening effect for postpartum LPS on the intensified Ang II vasoconstriction in male, but not female, offspring of PE rats, inferring a favorable postconditioning influence for LPS against renovascular perturbations induced by PE in male offspring. Indeed, the phenomenon of pre- or post-insult conditioning has been designated for LPS and other interventions against biological offences. For example, post-ischemic treatment with LPS was shown to reduce infarct volume following middle cerebral artery occlusion^55^. Similarly, post-insult hypothermia resulted in amelioration of hippocampal neurodegeneration in the rodent model of preterm oxygen deprivation^56^. Endotoxin preconditioning has also been credited in offsetting renal damage and ischemic stroke^57–60^. In these instances, LPS negatively regulates the inflammatory states through suppressing the activation of NFκB and proinflammatory genes^57,58^.

The upregulated RAS signaling is linked to inflammatory conditions such as hypertension and PE^61^. Ang II contributes to the pathophysiology of inflammatory tissue damage by promoting endothelial dysfunction, oxidative stress, and apoptosis^21^. Ang II is also believed to provoke nuclear NFκB translocation and subsequent expression of pro-inflammatory chemokines and cytokines^62^. Consistent with these reports, immunohistochemical studies undertaken in the present study revealed two important findings that establish a causal link between RAS and the sex-dependent PE/LPS interaction. First, there was the observation that the renal expressions of AT1 receptors and ACE, rate limiting enzyme in Ang II synthesis, were augmented in male offspring of PE dams and these effects were blunted in response to the postconditioning endotoxic challenge. These increases and decreases in renal ACE/AT1 signals evoked by PE and PE/LPS interventions, respectively, may logically explain the paralleled perturbations in renal Ang II vasoconstrictions in male offspring.

Unlike males, LPS postconditioning was not manifest in female offspring. The exaggerated renal vasoconstrictions induced by Ang II and phenylephrine and ACE/AT1 receptor overexpression were preserved in female offspring receiving the same challenging dose of LPS. Although the reason for this apparently sex-related PE/LPS interaction is not clear, a possible role for tubular ACE/ACE2 signaling in this regard cannot be overlooked. The increase in renal expression of ACE was less evident in female offspring. And, in contrast to no effect in male rats, an adaptive augmentation of ACE2 expression was observed in female renal tubules. The enhanced ACE2 expression may act via Ang1-7 generation and Ang II degradation^63^ to counterbalance the detrimental consequences set off by simultaneous overexpression of the offensive ACE/AT1 receptor arm. Ang1-7 is believed to possess antiinflammatory, antioxidative, and antithrombotic actions^64^. It is tempting to speculate that the abolition of the upregulated renal ACE2 signal in LPS-challenged female offspring of PE dams while maintaining the ACE/AT1 receptor expression may account for the augmented Ang II/phenylephrine vasoconstrictions.

To provide more insight into the role of the RAS-derived Ang1-7 in the PE/LPS interaction, experiments were conducted to investigate the effect of prenatal administration of Ang1-7 on renovascular and inflammatory responses in the current model system. The data showed that Ang1-7 was clearly more effective in normalizing functional and molecular renal profiles in female than in male offspring. This postulate is supported by the observations that (i) whereas phenylephrine vasoconstrictions were similarly attenuated by Ang1-7 in male and female offspring, Ang II responses were preferentially inhibited by the heptapeptide in female kidneys only, and (ii) the intensified renal AT1 receptor expression caused by PE or PE/LPS in female kidneys was offset by Ang1-7. These data implicate the Ang1-7-evoked downregulation of renal AT1 receptors in the reduced Ang II-mediated vasoconstriction in female offspring. Obviously, this contrast with the LPS postconditioning stimulus that failed to rectify renal anomalies sparked by PE in female offspring. These data are consistent with reports that the two peptide products of RAS, Ang II and Ang1-7, act through coordinated mechanisms to maintain homeostasis^65–67^.

The modulatory role of NFκB, an inducible transcription factor that regulates a large array of immune and inflammatory genes^57,58^, in the presumed renoprotective action of Ang1-7 cannot be overlooked. Our data showed that the reduced abundance of AT1 receptors caused by Ang1-7 in renal tissues of female offspring of PE/LPS rats coincided with remarkable suppression of the upregulated NFκB signal in the same model system. These observations are consistent with the established positive relationship between inflammatory pathways of Ang II and NFκB. Indeed, the nuclear translocation of NFκB translocation and subsequent expression of pro-inflammatory chemokines and cytokines are all facilitated by the RAS-derived Ang II^62^. Such depressant effect of Ang1-7 on tubular NFκB expression was not seen in male offspring of PE/LPS rats probably because of the NFκB signal was not upregulated in these rats compared with their female counterparts (see Fig. 8). Apparently, the demonstration of Ang1-7-mediated inhibition of NFκB expression appears to be dependent on the pre-existing level of this transcription factor.

It is imperative to comment on possible molecular mechanisms and clinical significance of endotoxic conditioning of the adverse effects caused by PE programming in male offspring. The novel genetic studies by Dia et al.^58^ suggested a pivotal role for miR146a, a key microRNA, in arbitrating endotoxic preconditioning of renal injury via suppressing the transcription of proinflammatory genes. Further, molecular and pharmacologic studies implicate heat shock protein 27 upregulation in the LPS-mediated renoprotection in a murine model of renal ischemia/reperfusion injury^60^. Evidently, these experimental observations highlight a therapeutic potential for LPS in renal injury. Likewise, findings of the current experimental study infer a possible advantageous action for LPS against exacerbated renal vasoconstriction and inflammation that appear in the adult life of preeclamptic offspring. Remarkably, the beneficial effects of the endotoxic challenge can arguably be demonstrated in two clinical settings. First, accidental rises in circulating endotoxin due possibly to infection or enhanced intestinal absorption of endotoxin may restrain the deteriorated renal sequels in adult offspring of PE mothers. Second, scheduled LPS challenge can be introduced to suppress existing renal toxicity in progenies of PE dams. The relevance of these clinical scenarios remains to be investigated.

The current study suffers at least two limitations. First, we reported here on the role of renal vasoconstrictor conduits in the PE/LPS interaction, and no attempt was made to determine whether the renal vasodilatory propensity exhibits a similar pattern. Indeed, imbalances in feedback and feedforward modulation of renal vasoconstrictor/vasodilatory profiles are believed to participate critically in renal microcirculation dysfunction observed during preeclamptic^68,69^ and endotoxic insults^70,71^. Another limitation of the current investigation was the focus on the offensive AT1 receptors, but not receptors of the defensive arm of RAS like MAS and AT2 receptors. The clinical studies by Chen et al.^72^ demonstrated that the renal expression of MAS receptors, endogenous binding sites for Ang1-7, is reduced in cultured preeclamptic glomerular podocytes and this effect was reversed after co-culturing with Ang1-7. The reduced availability of MAS receptors has been implicated in the provoked proteinuria and podocyte injury^72^. A paradoxical upsurge in MAS binding in uterine tissues of PE rodents was reported by Yamaleyeva et al.^73^ and suggested to serve as a compensatory mechanism for the rise in uteroplacental vascular resistance. The AT2 receptors, on the other hand, exhibit different expression patterns that depend on the gestational time and anatomical areas where the expression was assessed^73,74^. That said, no information is available regarding the roles of MAS and AT2 receptors in renal or extrarenal PE/LPS interaction. These two limitations provide a framework for future studies in our laboratory.

In conclusion, our data reveal sex-dependent effects for PE and endotoxic challenges on renovascular reactivity in adult offspring. On the one hand, the postconditioning effect of LPS on augmented renal vasoconstriction and upregulated ACE/AT1 receptor signals is featured in male, but not female, offspring of PE dams. To the contrary, a noticeable preconditioning renoprotection is conferred by gestational Ang1-7 supplementation in female offspring only. More studies are necessary to gain more insight into the potential roles of inflammatory, oxidative, and angiogenic pathways in this clinically relevant interaction.

## Materials and methods

### Animals

Adult Wistar rats (180–240 g) were obtained from the Animal facility of the Faculty of Pharmacy, Alexandria University, Egypt, maintained under controlled laboratory conditions and allowed free access to standard rat chow and tap water. All experimental protocols and animal manipulations were approved by the Institutional Animal Care and Use Committee, Alexandria University, Egypt (ACUC project, Approval No. AU06201957149, approval date. 7/5/2019), the "Principles of laboratory animal care" (NIH publication No. 86-23, revised 1985) were followed and were in accordance with the ARRIVE guidelines.

### PE induction

Pregnancy was prompted by allowing the mating between nulliparous adult female rats and larger male rats at a ratio 1:1. The day of conception was determined by the presence of a vaginal plug or spermatozoa in vaginal lavage. To induce PE in pregnant dams, N^ω^-nitro-l-arginine methyl ester (L-NAME) (50 mg/kg/day; Sigma-Aldrich Co, St. Louis, MO, USA) was administered via oral gavage for 7 consecutive days starting from day 14 of conception^3,75^. The development of PE was verified by the rises in SBP when measured on gestational day 20 from 111.9 ± 1.3 mmHg (non-PE rats) to 138.5 ± 1.9 mmHg (PE rats). Ten weeks after delivery, adult male and female offspring were processed for renal and hemodynamic assessments.

### The rat isolated perfused kidney

The procedure described in our previous studies^19,76,77^ has been used to assess the renal vasoconstrictor responsiveness to Ang II and phenylephrine. After the induction of anesthesia with i.p. thiopental (50 mg/kg; Biochemie, Vienna, Austria), the left kidney was exposed through a midline ventral laparotomy and the left renal artery was cannulated. The left kidney was then excised from surrounding tissue, rapidly mounted on a temperature-controlled glass chamber maintained at 37 °C, continuously perfused with Krebs’ solution (NaCl 120, KCl 5, CaCl_2_ 2.5, MgSO_4_⋅7H_2_O 1.2, KH_2_PO_4_ 1.2, NaHCO_3_ 25, and glucose 11 mM), maintained at 37 °C and gassed with 95% O_2_ and 5% CO_2_. The kidney was perfused at a constant flow rate of 5 ml/min by the means of a peristaltic pump (Model P3- Pharmacia Fine Chemicals). The perfusion pressure was continuously monitored by means of a BP transducer (Model P23XL, Astro-Med, Inc., West Warwick, RI, USA) connected to a computerized data acquisition system with LabChart-7 pro software (AD Instruments, Bella Vista, Australia). After a stabilization period of at least 30 min, cumulative dose response curves were established in each kidney by consecutive bolus injections of Ang II (0.25–32 ng; Sigma-Aldrich Co, St. Louis, MO, USA) and phenylephrine (0.41–900 ng; Sigma-Aldrich Co, St. Louis, MO, USA) into the perfusate line proximal to the kidney. Ang II and phenylephrine were prepared freshly in distilled water. Each dose of Ang II or phenylephrine was injected when the preceding dose has achieved its maximal vasoconstrictor response. Moreover, a wash period of 30 min was allowed in each kideny after the last dose of Ang II before establishing the phenylephrine curve. Values were expressed as changes in perfusion pressure from basal renal pressure.

### Tail-cuff plethysmography

Non-invasive SBP measurements for conscious pregnant rats and adult offspring were performed using the tail-cuff technique and a computerized data acquisition system with LabChart-7 pro software (Power Lab 4/30, Model ML866/P, AD Instruments, Bella Vista, Australia)^78^. All rats were subjected to daily pre-conditioning for at least 3 consecutive days before the actual measurement of SBP to get the rats adapted to measurement conditions. A tail cuff and pulse transducer (Pan Lab, Spain) were placed on the base of the tail. SBP measurement was carried out based on the periodic occlusion of tail blood flow. SBP was measured in triplicates and values were averaged.

### Immunohistochemistry

The protein expression of AT1 receptors, ACE, ACE2, and NFκB in rat renal tubular tissues was assessed according to method designated in our previous studies^38,79^. The right kidneys were fixed in 10% formaldehyde and embedded in paraffin blocks. Sections (4 µm thick) of kidney were cut and mounted on positively charged adhesive glass slides (Thermo Scientific, Berlin, Germany), then deparaffinized in xylene and rehydrated in a series of declining ethanol concentration (100, 95 and 70%). Heat-induced epitope retrieval was carried out by immersing slides in coplin jars containing 10 mM citrate buffer solution and incubated in a microwave at power 100 for 1 min then power 30 for 9 min. Endogenous peroxidases were blocked by 0.3% hydrogen peroxide for 10 min. The rabbit, anti-rat primary antibody for ACE (1 µg/µl, 1:300, Thermo Scientific, Berlin, Germany), ACE2 (1 µg/µl, 1:300, Thermo Scientific, Berlin, Germany), AT1 (1 µg/µl, 1:300, Thermo Scientific, Berlin, Germany), and NFκB p65 (1 µg/µl, 1:200, Thermo Fisher Scientific, Waltham, Massachusetts, USA) were applied to the slides and then sections were incubated at 4 °C overnight. The secondary antibody horseradish peroxidase was applied for 30 min. The chromogen 3,3′-diaminobenzidine was prepared and applied as instructed by the manufacturer for protein visualization. Slides were counterstained with hematoxylin and dipped in ascending concentrations of alcohol and then xylene. Images of renal tubular tissues were taken by OptikamB9 digital camera (Optika Microscopes, Italy). Fiji Image J Software Version 1.51n (National Institutes of Health, Bethesda, Maryland, USA) was used to measure the percentage of chromogen 3,3′-diaminobenzidine positive stained area in the tubular cortex, outer medullary areas.

### Protocols and experimental groups

This experiment was performed to investigate the effect of PE fetal programming on hemodynamic, renovascular and inflammatory consequences of endotoxemia in male and female offspring of PE and non-PE mothers. Eight adult offspring rat groups were used (10 weeks old, n = 7–8 each). For each gender, 4 groups of rats were employed and categorized into: (i) saline-treated non-PE offspring, (ii) LPS-treated non-PE offspring, (iii) saline-treated PE offspring, and (iv) LPS-treated PE offspring. On the experiment day, endotoxemia was induced in adult offspring by i.p. injection of a 5 mg/kg dose of LPS (from E coli, serotype 0111:B4; Sigma-Aldrich Co, St. Louis, MO, USA)^19^. LPS was dissolved in saline. Four hours later, SBP was measured using tail cuff plethysmography and rats were then anesthetized and left kidneys were isolated and perfused to evaluate vasoconstrictor responsiveness to Ang II and phenylephrine. Additionally, right kidneys were collected for immunohistochemical determination of AT1, ACE, ACE 2 and NFκB protein expressions in renal tubular tissues.

Another experiment was performed to determine whether prenatal therapy with Ang1-7 would modify hemodynamic and renal damages caused by the PE/LPS interaction in adult offspring. Ang1-7 (576 µg/kg/day, s.c.; Sigma-Aldrich Co, St. Louis, MO, USA)^80^ was administered to pregnant rats along with L-NAME (50 mg/kg/day) for 7 consecutive days, starting from gestational day 14. Ten weeks after spontaneous delivery, LPS (5 mg/kg i.p.) was administered to male and female offspring (n = 7–8 each) and 4 h later, SBP measurement, renovascular and immunhistochemical studies were performed as described above.

### Statistical analysis

Data are expressed as means ± S.E.M. In isolated perfused kidneys, vasoconstrictor responses to Ang II and phenylephrine were expressed as changes from basal perfusion pressure. In immunohistochemical studies, the percentages of stained areas were estimated. Normal distribution was checked using column statistics (Shapiro–Wilk Normality Test, GraphPad Prism, software release 8.0.2). The one-way ANOVA followed by the Tukey's post hoc test was used to assess statistical significance with probability levels < 0.05.

## Supplementary Information

Supplementary Information.

## Data Availability

Raw data are provided as an additional supporting file.
